# Outcomes of early endoscopic realignment for blunt straddle injuries to the bulbar urethra: a single-center retrospective study

**DOI:** 10.1186/s12893-022-01489-z

**Published:** 2022-01-29

**Authors:** Jianwei Wang, Zhengqing Bao, Xiao Xu, Zhenhua Liu, Guizhong Li, Guanglin Huang, Libo Man

**Affiliations:** Urology Department, Beijing Jishuitan Hospital, The Fourth Medical College of Peking University, Beijing, China

**Keywords:** Urethra, Urethral obstruction, Wounds and injuries, Crush injuries, Minimally invasive surgery

## Abstract

**Background:**

The optimal acute management of patients with blunt straddle injury to the bulbar urethra remains in question. Conventionally, suprapubic diversion with delayed urethroplasty can always be considered, if necessary, but the role of early endoscopic realignment (EER) in the acute management of blunt straddle injuries to bulbar urethra is controversial. We report our clinical experience and outcomes with EER for patients with straddle injury to the bulbar urethra in a level one trauma center.

**Methods:**

We retrospectively reviewed 44 male patients who were transferred to our trauma center between January 2013 and January 2019 for acute management of blunt straddle injury to the perineum leading to bulbar urethra injuries. We reviewed the medical records of those patients to identify demographics, emergency management and clinical outcomes.

**Results:**

The most common injury mechanism was falling onto the perineum (n = 27, 61.4%), followed by motorcycle accident (n = 11, 25.0%) and bicycle accident (n = 6, 13.6%). Of the 44 patients, 14 (31.8%) were partial bulbar urethral ruptures and 30 (68.2%) were complete bulbar urethral ruptures. 31 (70.5%) patients successfully underwent EER and 13 (29.5%) patients failed attempted EER. the difference between successful EER attempts and failed ones in term of injured urethral mucosa integrity was statistically significant (*P* = 0.035, OR 8.667,95% CI: 0.998–75.235). In patients who underwent successful EER, urethral stricture occurred after catheter removal at a median of 8 (1–28) months in 24 (77.4%) patients and the mean stricture length was 1.8 ± 0.8 (0.5–3.0), which was not statistically significant when compared with those who failed EER (*P* = 0.103). Overall, 21 out of 24 (87.5%) patients with strictures after EER were successfully managed by urethroplasty.

**Conclusions:**

Although achieving a successful EER attempt is relatively easy for most patients with straddle injury to the bulbar urethra, it does not improve urethral healing significantly. Most patients with stricture formation after EER have to be cured with urethroplasty.

## Background

Bulbar urethral injury usually occurs as a result of blunt straddle injury and is more common than posterior urethral injury [1]. The optimal acute management of patients with blunt straddle injury remains in question [2–4]. Immediate operative intervention to repair or debride the injured urethra is contraindicated due to the indistinct nature of the injury border [5]. Clinicians should promptly establish urinary drainage in patients with straddle injury to the bulbar urethra to minimize urinary extravasation [5, 6]. If one gentle attempt at blind urethral catheterization has failed, the acute therapeutic options include suprapubic diversion or attempted early endoscopic realignment (EER) are needed [7].

Conventionally, suprapubic diversion with delayed urethroplasty can always be considered for patients with anterior urethral injuries, if necessary. The advancement in endoscopy has led to EER emerging as a rival minimally invasive treatment option for patients with PFUI [8, 9], but the role of EER in the acute management of blunt straddle injuries is controversial [2, 6, 9–11]. We herein report our experience with EER for acute management of blunt straddle injuries to the bulbar urethra in a level one trauma center in China.

## Methods

### Patients

**Fig. 1:**
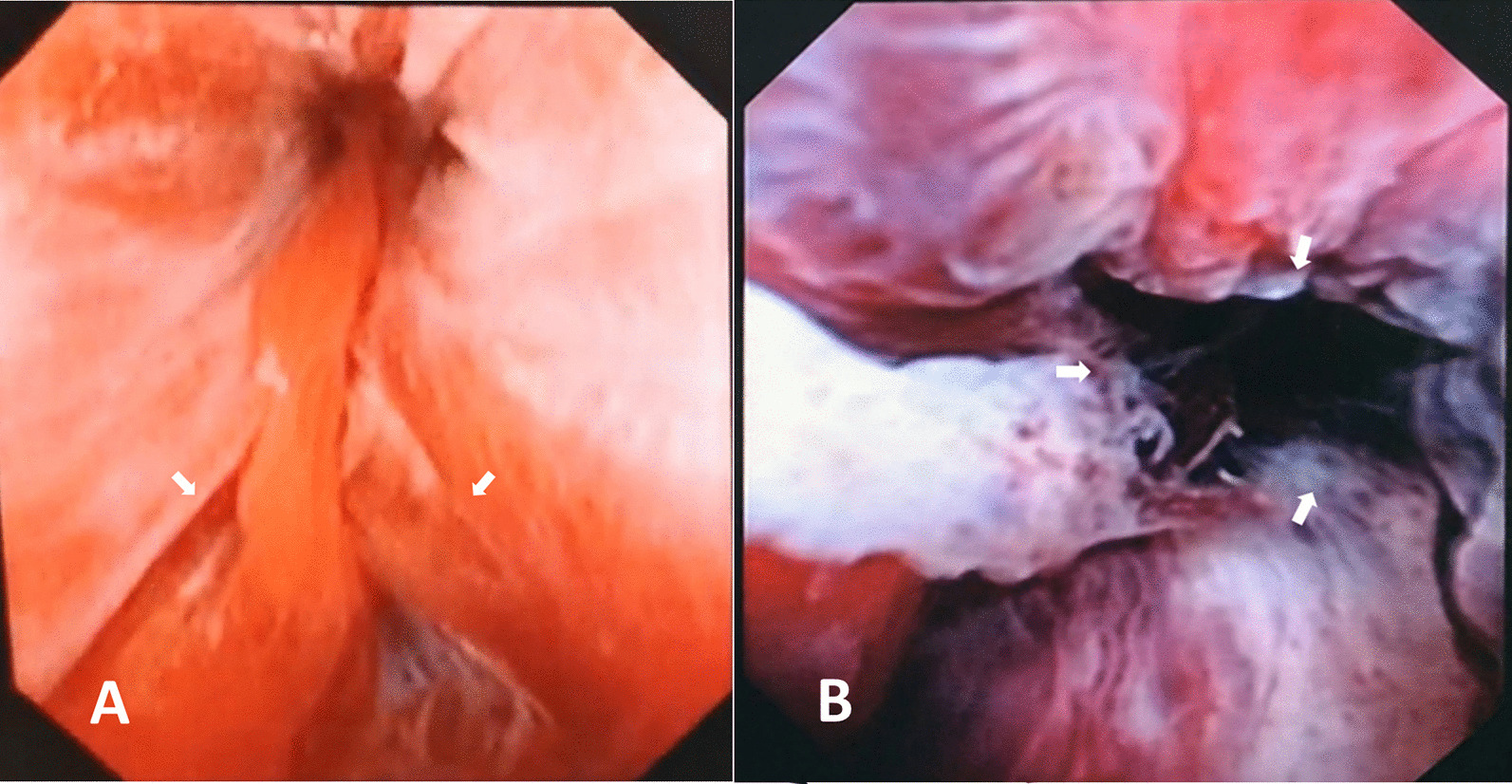
Staging of the injury with findings of urethrocystoscopy. **A** Partial rupture; **B** Complete rupture. White arrows show the margins of the broken urethal mucosa

After obtaining permission from our institutional ethical committee, we retrospectively reviewed 54 male patients who were transferred to our trauma center between January 2013 and January 2019 with blunt straddle injury to the perineum and acutely managed by EER at Beijing Jishuitan hospital, which is the trauma center of Beijing and a specialized center for urethral reconstruction. There were 44 male patients after excluding those with incomplete clinical data, concomitant pelvic bone fracture or lost to follow-up. We reviewed the medical records to identify demographics, emergency management, and clinical outcomes. The traumatic urethral injuries were assigned to two groups, partial rupture and complete rupture, based on the integrity of the urethral mucosa observed by urethrocystoscopy [Fig. 1]. All EER were performed by two urethral reconstructive urologists in our center.

### Surgical techniques

After the suspicion of bulbar urethral injury, we routinely underwent a gentle attempt at blind urethral catheterization in the emergency department then followed with an attempt of EER when it failed unless there were unstable vital signs. In cases with partial rupture, a retrograde urethrocystoscopy was performed with a flexible cystoscope to bypass the ruptured segment and introduce a guide wire through its channel to the bladder, which facilitated placement of a silicone catheter (F16) by a Seldinger technique [12]. If this procedure failed or for patients with complete rupture, the suprapubic catheterization (SPC) could be placed transcutaneously under ultrasound guidance. When the bladder was not distended enough for safe puncture, we placed a needle into the bladder under ultrasound guidance and instilled additional saline to assist percutaneous puncture. An antegrade cystoscopy was performed through the SPT to find the bladder neck before negotiating it to the proximal side of the ruptured segment. A guide wire was introduced by the flexible cystoscope channel into the ruptured segment. Then, a retrograde urethrocystoscopy was performed to find the tip of the guide wire and grasp it with forceps. The guide wire was pulled out of the meatus, followed by placement of a silicone catheter (F16) by a Seldinger technique. If the time of the operation was longer than 60 min or the flexible cystoscopic maneuver was longer than 30 min, and it was nont possible to find the tip of the guide wire with the retrograde urethrocystoscopy, we regarded it as a failure and patients were maintained on SPC drainage to await delayed management.

### Follow-up

We removed the urethral catheter 4 weeks later. Success was defined as the patient having no obstructive symptoms and requiring no further treatment. Patients were followed up postoperatively at 1, 3, 6, and 12 months and annually thereafter. A flexible urethrocystoscopy and retrograde urehtrography were performed when recurrent stricture was suspected. The primary outcome is the development of urethral stricture.

### Statistical analysis

Continuous data are presented as mean ± SD or median with ranges, and categorical data are presented as frequencies and percentages. Student’s t-test or the Mann–Whitney U test was used for continuous data to evaluate comparisons between the groups. The chi-square test was used for categorical data. All p-values were 2-tailed, and p < 0.05 was considered statistically significant. Statistical analyses were performed by using SPSS ver.19.0 (SPSS Inc., Chicago,IL, USA).

## Results

Patient characteristics are listed in Table 1. The mean age was 39.7 ± 16.1 (range, 16–71) years. The most common injury mechanism was falling onto the perineum (n = 27, 61.4%), followed by motorcycle accident (n = 11, 25.0%) and bicycle accident (n = 6, 13.6%). 10 (22.7%) patients had concomitant perineal hematomas and 9 (20.0%) had scrotal hematomas. Of the 44 patients, 14 (31.8%) were partial rupture and 30 (68.2%) were complete rupture. Of the 44 patients, 19 (43.2%) of injuries were located in the distal or mid bulbar urethra and 25 (56.8%) in the proximal bulbar urethra, in which 5 (11.4%) involved the membranous urethra.

**Table 1:** Patient characteristics

Characteristic	Total	Partial rupture	Complete rupture	*P value*	OR (95% CI)
Number of patients	44	14 (31.8)	30 (68.2)		
Age, mean and range, y	39.7±16.1 (16–71)	38.2±17.0 (20–71)	40.4±15.9 (16–70)	0.676	
Injury mechanism, n (%)					
Fall	27 (61.4)	8	19		
Motorcycle accident	11 (25.0)	4	7		
Bicycle accident	6 (13.6)	2	4	0.918	
Concomitant injures, n (%)					
Scrotal hematoma	9 (20.0)	2	7		
Perineal hematoma	10 (22.7)	3	7	1.000	0.667 (0.084–5.301)

**Fig. 2:**
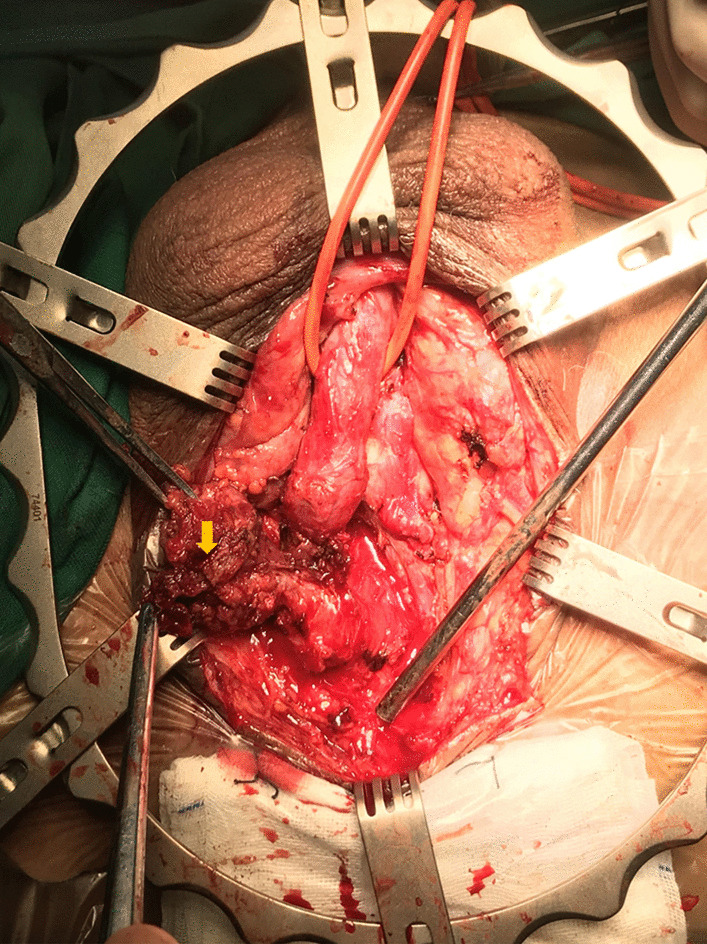
Perineal abscess developed after EER. The yellow arrow indicates the perineal abscess and the necrotic tissue around it

The mean duration from injury to EER was 11.4 ± 9.0 (2–48) hours, and 41 (93.2%) cases underwent EER within 24 h (except 3 patients who were referred to our center after initial SPC placement in other hospitals). Detailed surgery information and outcomes of EER is provided in Table 2. The mean operating time was 33.9 ± 10.8 (10–60) minutes and 24 (54.5%) cases were completed within 30 min. 31 (70.5%) patients successfully underwent EER and 13 (29.5%) patients failed EER attempt. Those patients who failed attempted EER were maintained on SPC drainage and underwent delayed urethroplasty at 3 months later. Of 31 patients who underwent successful EER, 13 (13/14, 92.9%) were patients with partial rupture and 18 (18/30, 60.0%) were complete rupture. The difference between successful EER attempts and failed ones in term of injured urethral mucosa integrity was statistically significant (*P* = 0.035, OR 8.667, 95% CI 0.998–75.235). One patient with failed EER attempt developed a local perineal abscess postoperatively that was found during urethroplasty and the patient had an uneventful healing after en bloc removal of the perineal abscess and anastomotic urethroplasty [Fig. 2]; Two patients developed epididymitis and were successfully managed with antibiotic therapy.

**Table 2:** Surgical outcomes

Parameters	Total	Partial rupture	Complete rupture	*P *value	OR (95% CI)
Time from injury to EER, mean and range, h	11.4±9.0 (2–48)	8.1±3.7 (2–14)	13.0±10.3 (4–48)	0.092	
Lesion location to bulbar urethra, n (%)					
Distal or middle	19 (43.2)	8	11		
Proximal	25 (56.8)	6	19	1.000	1.091 (0.275-4.324)
Membranous urethral involved	5 (11.4)	0	5		
Operation time, mean and range, min	33.9±10.8 (10–60)	28.2±9.5 (10–40)	36.5±10.4 (20–60)	0.016	
Perioperative complication, n (%)					
Periurethral abscess	1 (2.3)	0	1		
Epididymitis	2 (4.5)	1	1		
Bleeding	7 (15.9)	1	6		
Surgical outcome, n (%)					
Successful EER attempt	31 (70.5)	13 (92.9)	18 (60.0)		
Failed EER attempt	13 (29.5)	1	12	0.035	8.667 (0.998–75.235)
Outcomes of Patients with successful EER attempt, n (%)					
Successful	7/31 (22.6)	5	2		
Failed	24/31 (77.4)	8	16	0.099	0.200 (0.032–1.267)
Follow-up, median and range, mo	37.5 (12–78)	32.5 (12-63)	38.0 (12–78)	0.000	
Time to spontaneous voiding, mean and range, mo	4.2±3.1 (1–12)	4.0±2.9 (1–11)	4.3±3.3 (1–12)	0.795	
Stricture length, mean and range, n (%)					
Failed EER	2.2±0.6 (1.0–3.0)				
Occurred after successful EER	1.8±0.8 (0.5–3.0			0.103

The median follow-up was 37.5 (12–78) months. The mean time to spontaneous voiding was 4.2 ± 3.1 (1–12) months. 79.5% of patients were voiding naturally within 6 months. All patients with failed EER attempt underwent delayed urethroplasty three months later and the mean stricture length was 2.2 ± 0.6 (1.0–3.0)cm. Postoperatively, one case of recurrent stricture was diagnosed by urethrocystoscopy and was managed successfully with dilation. In patients with successful EER attempt, urethral stricture occurred after removal of the catheter at a median of 8 (1–28) months in 24 (77.4%) patients and the mean stricture length was 1.8 ± 0.8 (0.5–3.0), which was not statistically significant when compared with those of failed EER (*P* = 0.103). 4 (16.7%) patients with urethral stricture were treated with dilation, 4 (16.7%) with DVIU and 16 (66.7%) with urethroplasty. Two cases of failed dilation and 3 cases of failed DVIU were successfully managed with urethroplasty. Overall, 21 out of 24 (87.5%) patients with strictures after EER were successfully managed by urethroplasty. De novo erectile dysfunction occurred in 3 (6.8%) cases. There were no cases of de novo incontinence.

## Discussion

After blunt straddle injury to the perineum, the primary morbidity is anterior urethral stricture [13]. Blunt straddle injury is associated with spongiosal contusion, which makes it more difficult to discern the limits of urethral debridement and define the accurate anatomy of adjacent structures, thus acute or early urethroplastly is not indicated [14]. Clinicians should initially establish prompt urinary drainage in acutely injured patients with straddle injury to the anterior urethra in order to prevent urinary extravasation and infection [2, 5]. Therapeutic options for blunt straddle injury include suprapubic diversion or attempted early endoscopic realignment [15]. Both EAU and AUA recommend suprapubic or urethral catheter placement and delayed treatment for blunt trauma to the anterior urethra because the extent of injury is hard to evaluate [16]. Due to the relatively low incidence of urethral injuries and limited available evidence, there is controversy surrounding the optimal management strategy for blunt straddle injury to the bulbar urethra [2–4].

Very few literatures mentioned the success rate of EER for anterior urethral injury. Hadjizacharia et al. [11] reported eighteen patients with acute urethral injuries who underwent endoscopic realignment attempt. There were 6 cases of patients with bulbar urethral injuries and the successful rate of endoscopic realignment attempt was 50%. In our report, 31 (70.5%) patients successfully underwent EER attempt and 13 (29.5%) failed the procedure mainly due to the severity of their injury, and some of them were stopped because the time limits of flexible cystoscopic manipulation was reached. Many reasons may explain the failure of EER attempt including the degree of the injury and the experience of the surgeon. For those with successful EER attempts, the percentage of patients with partial rupture (13/14, 92.9%) is higher than those with total disruption (18/30, 60.0%). Also, the difference between successful EER attempts and failed ones in term of injured urethral mucosa integrity was statistically significant (*P* = 0.035).

For better vision during EER, irrigation with pressure is needed, which may increase the chance of infection and cause additional injury to the ruptured urethra along with flexible urethroscopic manipulation during endoscopic realignment [4]. Although we limited the flexible cystoscopic manipulation time, the impact of procedure related injury and perioperative infection should be followed. Early reports by Elgammal MA, et al. [4] stated that in cases of complete urethral injury with perineal hematoma or extravasation, no attempt at urethral realignment was made. However, our study showed although 10 (23.7%) patients had concomitant perineal hematomas and 9 (20.0%) had scrotal hematomas, only 1 case of perineal abscess developed after EER, which was found during surgical exploration and healed uneventfully with treatment. Possible reasons for our good surgical outcomes include broad-spectrum antibiotic use perioperatively and our adherence to strict time limitations for endoscopic manipulating. Also, 2 cases of postoperative epididymitis were successfully managed with antibiotics. Although infection was one of the major complications of EER in our series, there was no significant negative effect on surgical outcomes of urethroplasty.

There is an assumption that with early urine diversion for total or partial urethral disruption the extent of the acute and chronic inflammatory cascade would be limited and the severity of residual stricture may be mitigated [13]. Also, research suggests that a urethral catheter placed across an injured urethra by primary realignment might augment appropriate urethral healing and subsequently decrease urethral stricture rate [2]. Seo et al. [3] reported the long-term outcome of primary endoscopic realignment for bulbar urethral injuries and 39.2% patients developed urethral strictures in 89.1 ± 36.6 months after surgery. Elgammal et al. [4] retrospectively studied the management and outcome in 53 patients with straddle injuries to the bulbar urethra. Strictures occurred in 11 of 31 (35%) patients treated initially with SPC and in 18 of 22 (82%) treated with primary urethral realignment (p < 0.01). Park’s research showed that mean stricture length of patients with blunt straddle injuries to the anterior urethra was significantly longer in men with delayed presentation (2.7 vs. 1.8 cm, p < 0.05) [13]. It may be the prolonged urine extravasation into the spongiosum that leads to more spongiofibrosis and greater stricture length. The results of our report showed that strictures occurred in 24 of 31 (77.4%) patients treated initially with successful attempted EER, and the mean stricture length was 1.8 ± 0.8 (0.5–3.0), which was shorter than those with failed EER. However, there is no statistically significant difference when comparing the length of urethral strictures in patients after successful EER to those with failed EER (*P* = 0.103). Furthermore, patients with relatively mild injury such as a direct kick to the perineal regions might have more chance to achieve a successful catheterization when performing an attempt of gentle blind urethral catheterization and be followed with a good healing, which are not included in our series. That might explain why the rate of urethral strictures is higher when compared to other studies [3, 11]. Thus, the impact of EER on the healing of the injured bulbar urethra and the development of late urethral stricture should the subject of further investigation.

Controversy remains regarding whether SPC or EER is the better acute management strategy for patients with straddle injuries. Although EER is a minimally invasive treatment for patients with blunt straddle injury in an acute setting and is technically possible to perform in most patients, the stricture formation after EER does not decrease significantly. Indeed, 87.5% (21/24) of patients with stricture formation after successful EER attempt require urethroplasty, which is the same endpoint for patients treated with SPC alone. Conventionally, performing a urethroscopy with an attempt of EER in acute setting is a routine procedure in our center for patients with EER. A suprapubic diversion as the initial management should be recommend for some patients with straddle injury because the rate of urethral stricture formation is still very high even in patients with successful EER whether with partial or total urethral rupture according to the findings of our study. Actually, the acute management of straddle injury is shifting from EER to directly performing suprapubic diversion and waiting for later urethroplasty in our present emergent practice, especially for those with total rupture.

In our series, the endoscopic interventions, including dilation and DVIU, for urethral strictures after EER brought patients with a low success rate, and most of them were successfully managed by urethroplasty instead of dilation or DVIU again. According to the AUA guideline for male urethral stricture, another endoscopic procedure is unlikely to be successful for patients who are previously treated with dilation of DVIU because repeated endoscopic treatment may cause longer strictures and also increase the complexity of subsequent urethroplasty [17].

This study has several limitations. First, the sample size of 44 is large than most of the prior reports, but may still be small. For example, the lengths of stricture between successful and failed EER may become significant (p = 0.109 in our study) in a study of large size. Second, the follow-up time of 3 years could be longer to reveal the long-term outcome of EER. Finally, this retrospective study may have some selection and recall biases. Additional works are needed to address these limitations.

## Conclusions

Although achieving a successful EER attempt is relatively easy for most patients with straddle injury to the bulbar urethra, it does not improve urethral healing significantly. Most patients with stricture formation after EER have to be cured with urethroplasty.

## Data Availability

The datasets used and/or analyzed during the current study are available from the corresponding author on reasonable request.

## Abbreviations

EER: Early endoscopic realignment
PFUI: Pelvic fracture urethral injury
SPC: Suprapubic catheterization
EAU: European Association of Urology
AUA: American Urology Association
